# Antimicrobial activities of *Tephrosia vogelii* against selected pathogenic fungi and bacteria strains

**DOI:** 10.1080/21501203.2019.1705929

**Published:** 2019-12-19

**Authors:** Stephano Hanolo Mlozi, Juma A. Mmongoyo, Musa Chacha

**Affiliations:** a School of Life Science and Bioengineering, Nelson Mandela African Institution of Science and Technology, Arusha, Tanzania; b Department of Chemistry, University of Dar es Salaam, Mkwawa University College of Education, Iringa, Tanzania

**Keywords:** *Tephrosia vogelii*, antifungal agents, antibacterial agents, methanolic extracts

## Abstract

*Candida albicans* and *Cryptococcus neoformans* are dangerous pathogens causing fungal diseases. *C. albicans* and *C. neoformans* developed resistance to fungicides such as fluconazole. Similarly, pathogenic bacteria *Staphylococcus aureus, Escherichia coli, Klebsiella pneumoniae and Salmonella typhi* have become resistant to antibiotcs such as methicillin. Thus, searching for alternative antimicrobial agents is inevitable. *Tephrosia vogelii* used traditionally for management of fungal and bacterial diseases is potential source of antimicrobial agents. It is in this vein that, antimicrobial activities of leaf and root extracts of *T. vogelii* were evaluated against *C. albicans* (ATCC 90028), *C. neoformans* (clinical isolate), *S. aureus* (ATCC25923), *E. coli* (ATCC29953), *K. pneumoniae* (ATCC 700603) and *S. typhi* (NCTC 8385). A two-fold serial dilution method using the sterilised 96 wells of polystyrene microlitre plates used to determine the minimum inhibitory concentration (MIC) of extracts. Hexane and dichloromethane extracts exhibited the lowest activity against fungi strains with MICs >10 mg/mL. Root and leaf methanolic extracts exhibited activity at MICs of 5 and 1.25 mg/mL, respectively, against both tested fungi. Dichloromethane and methanolic extracts exhibited antibacterial activity with MICs ranging from 2.5 - 10 mg/mL and 0.625 - 5 mg/mL, respectively. Antimicrobial activities of the extracts of *T. vogelii* revealed potentiality of bioactives against fungal and bacterial diseases.

## Introduction

Fungal diseases have been reported to affect over a billion people worldwide whereby more than 1.5 million people die of such diseases every year (Bongomin et al. 2017). The *Candida albicans* and *Cryptococcus neoformans* significantly contribute to deadly fungal infections to humans (Bongomin et al. 2017; Rodrigues and Albuquerque 2018). The *Candida albicans* and *Cryptococcus neoformans* are opportunistic fungi, which cause fungal life-threatening diseases: skin infections, oral infections, lung infections and central nervous system (CDC 2019). The climate change exacerbates the prevalence of fungal diseases and the emergence of such pathogenic fungi worldwide (Garcia-solache and Casadevall 2010). The immune-suppressed people living with cancer, diabetes and HIV/AIDS are tremendously vulnerable to opportunistic *Candida albicans* and *Cryptococcus neoformans* (Faini et al. 2015). The synthetic fungal drugs such as fluconazole are increasingly becoming less effective due to resistance developed by the pathogenic fungi (Vandeputte et al. 2012; Arendrup 2014; Rodrigues and Albuquerque 2018).

Nevertheless, little attention is paid to fungal diseases hence resulting in fewer efforts in searching for potential alternative antifungal agents against these opportunistic fungi which cause life-threatening fungal diseases (Bongomin et al. 2017; Rodrigues and Albuquerque 2018). Despite their life-threatening effects, unfortunately, these fungal diseases are often neglected. Consequently, the magnitude of the problem has become even more severe particularly now when the global statistics of the vulnerable immuno-suppressed people have considerably continued to rise (Faini et al. 2015). Therefore, a search for alternative antifungal agents against *Candida albicans* and *Cryptococcus neoformans* is obligatory and urgently needed (Rodrigues and Albuquerque 2018).

On the other hand, the pathogenic bacteria *Staphylococcus aureus, Escherichia coli, Klebsiella pneumoniae* and *Salmonella typhi* have developed resistance against the first-line antibacterial agents (Okeke et al. 2007; Labar et al. 2012). For instance, *Staphylococcus aureus* is a global problem due to its antibacterial resistance known as methicillin resistance *Staphylococcus aureus* (MRSA) (Okeke et al. 2007; Labar et al. 2012). Antibacterial resistance is dangerous because it makes control of infectious diseases difficult thereby leading to increased morbidity and mortality (Okeke et al., 2007). Also, such resistance imposes high costs on societies to deal with the bacterial pathogens (Okeke et al., 2007). This necessitates a search for new antibacterial agents capable of offsetting the bacterial resistances to promote public health.

Traditionally, medicinal plants are promising natural resources against fungal and bacterial diseases as they have been used since medieval times for management and treatment of human diseases (Maregesi et al. 2007; Tabassum and Hamdani 2014; Masevhe et al. 2015). Up to date, about 80% of human population still depend on medicinal plants to cure human ailments worldwide (Gurib-Fakim 2006). For instance, in Tanzania, thousands of medicinal plant species are traditionally used for the treatment of animal and human diseases including fungal and bacterial ailments (Hamza et al. 2006; Maregesi et al. 2007). Therefore, this study intends to search for potential antimicrobial agents from *Tephrosia vogelii* Hook. F. leaves and roots. Of particular interest from ethnomedical viewpoint, studies have shown that *Tephrosia vogelii* has traditionally been used not only as abortifacient and purgative agents but as agents for the management of ecto-parasitism, and schistosomiasis (Dzenda et al. 2008; Orwa et al. 2009). It is also used for the treatment of ringworms and skin diseases while its decoctions have been reported to cure scabies, yaws and constipation (Orwa et al. 2009). Pharmacological studies have also shown that extracts of *T. vogelii* leaves have substantial potency bioactives against lice, mites, fleas, ticks and mosquito larvicidal (Anjarwalla et al. 2015; Kidukuli et al. 2015; Stevenson and Belmain 2017). These ethnopharmacological reports appear to be adequate evidence that *T. vogelii* may be a promising source of novel antifungal and antibacterial agents which could offset the issue of fungal and bacterial resistance experienced by the modern synthetic antifungal and antibacterial drugs against the selected pathogenic fungi and bacteria.

In spite of ethnopharmacological evidence, there is limited information on the antifungal potential of *T. vogelii* against *C. albicans* and *C. neoformans* pathogens. Similarly, to the best of our knowledge, there is limited report on antibacterial potential of *T. vogelii* against *Staphylococcus aureus, Escherichia coli, Klebsiella pneumoniae* and *Salmonella typhi*. Thus, this study aims to investigate the antimicrobial potential of *T. vogelii* as a new strategy and trajectory to fight fungal and bacterial diseases; and to offset antimicrobial resistances.

## Materials and methods

### Chemicals

All chemicals and solvents used; methanol, dichloromethane, *n*-hexane and dimethyl sulphoxide (DMSO) were purchased from Sigma-Aldrich and used as they are.

### Plant materials, chemicals and extractions

The leaves and roots of *Tephrosia vogelii* were collected in January 2019 from Hai district, Moshi region in Tanzania (Latitude S 03° 15ʹ 6.4ʹ’ and longitude E 37° 14ʹ 3.8ʹ’). The plant species was identified by Mr Ezekiel John, the plant taxonomist and botanist from Tanzania Pesticides Research Institute (TPRI), and the voucher specimen (SH-NM102) was deposited at the Nelson Mandela African Institution of Science and Technology (NM-AIST). The plant belongs to phylum Tracheophyta, order Fabales, class Magnoliopsida, family Fabaceae, genus *Tephrosia* and species *Tephrosia vogelii*. The collected plant materials were air-dried under shade for 4 weeks then pulverised to powder form for subsequent extraction.

Chromatographic techniques were employed for extraction processes. The pulverised 0.6 kg leaves and 0.45 kg roots were extracted separately and sequentially soaked with n-hexane, dichloromethane, and methanol for 48 h in each solvent. All solvents were completely evaporated under low pressure using a rotary evaporator at a temperature below 40°C to avoid thermal decomposition of volatile compounds. After evaporation, 1.8 and 1.6 g of *n*-hexane leaf and root extracts, respectively, were obtained. Also, 2.0 and 1.5 g of dichloromethane leaf and root extracts, respectively, were obtained. In the same way, 12.0 and 5 g of methanolic leaf and root extracts, respectively were obtained. Then, the leaf and root extracts of *T. vogelii* (Table 1) were stored at 4°C for subsequent bioassays.

### Fungi strains, bacteria strains and sub-culturing

All strains used in this study were generously provided by the School of Pharmacy at Muhimbili University of Health and Allied Sciences (MUHAS). All bioassays were performed in the Microbiology Laboratory at MUHAS. Two fungal strains used were *Candida albicans* (ATCC 90028) and *Cryptococcus neoformans* (clinical isolate). Four bacteria strains were of two categories: the Gram positive bacteria, *Staphylococcus aureus* (ATCC25923) and the Gram negative bacteria; *Escherichia coli* (ATCC29953), *Klebsiella pneumoniae* (ATCC 700603) and *Salmonella typhi* (NCTC 8385). All microbes, fungi and bacteria were sub-cultured onto Mueller Hinton Broth (MHB). The MHB (8.0 g) was suspended in 230 mL of distilled water in 500 mL scotch bottle forming the mixture that was heated at 60°C to dissolve the agar completely. Then, the suspension was autoclaved at 121°C for 15 min. The mixture was left to cool at room temperature; inoculation was conducted onto the cooled growth media. Inoculation of strains was carried out then followed by incubation. The fungi strains inoculums were incubated at 37°C for 48 h while bacteria inoculums were incubated at 37°C for 24 h.

### Antimicrobial activities

**Figure 1.**
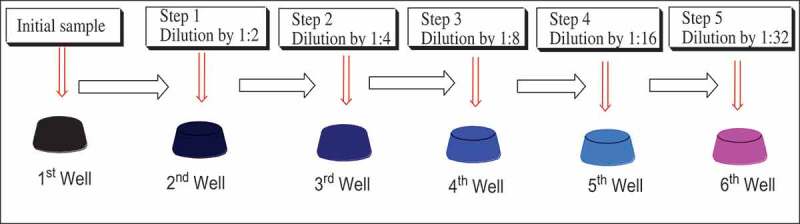
The concept of serial dilution.

The antifungal and antibacterial bioassays were conducted against *Candida albicans, Cryptococcus neoformans, Staphylococcus aureus, Escherichia coli, Klebsiella pneumoniae* and *Salmonella typhi* using methanolic, dichloromethane and *n-*hexane leaf extracts and root extracts of *T. vogelii*. The Mueller Hinton Broth (MHB) was used as sub-culture media in the experimental study. Two-fold serial dilution method (Figure 1) was used to determine the Minimal Inhibitory Concentration (MIC) of the extracts. This method was performed using the sterilised 96 wells of polystyrene microlitre plates as previously described with minor modifications (Mwangomo et al. 2012; Mlozi et al. 2017).

### Determination of minimum inhibitory concentration

The Minimal Inhibitory Concentration (MIC) values were determined by the two-fold serial dilution method. Forty milligrams of each crude extract was used to prepare stock solutions (40 mg/mL) by dissolving 40 mg of crude extracts in 1 mL of 10% DMSO and 90% sterile tryptone broth in eppendorf tube. The turbidity of inoculated microbes (fungi and bacteria) grown into MHB cultures for screening were adjusted to standard solution which is equivalent to the 0.5 McFarland units (Approximately 1.2 × 10^8^ CFU/mL). The inoculums were then subjected to biological assays.

The MHB (50 μL) were added to each microlitre plate well. Then, to each well of the first row, 50 μL of the extract (40 mg/mL) were added and well mixed to reduce the extract concentration to 20 mg/mL. The serial dilution proceeded by transferring 50 μL of the mixture from each well of the first row to each well in the second row. This dilution was carried on to the subsequent rows until the last row of the wells. The mixture (50 μL) transferred from each well of the last row were discarded. Then, 50 μL of the cultured strains (fungi and bacteria) were added to each well containing growth media poisoned with the extract completing the double dilution producing eight serial concentrations: 10, 5, 2.5, 1.25, 0.625, 0.3125, 0.1562, and 0.07825 mg/mL. The microlitre plate wells having inoculums without extracts were used as growth control (negative control). The microlitre plate wells with inoculums treated with fluconazole and ciprofloxacin were used as a positive control for fungi and bacteria, respectively. Then, all inoculated microlitre plate wells were incubated for 48 and 24 h for fungi and bacteria strains, respectively. After incubation, 30 μL of Iodonitrotetrazolium (INT) chloride salt was added to each well and then the plates were incubated for 1 hr, the duration after which the results were recorded. Whereas the pink colouration developed in microplate wells after addition of INT indicated the presence of microbes, clearance or disappearance of pink colouration indicated the growth inhibition of the fungi or bacteria. The MIC values of all extracts were determined at the lowest concentration of the extract at which a marked reduction in colour formation was noted. The negative control was used as reference for the growth of the strains under study.

## Results and discussion

**Figure 2.**
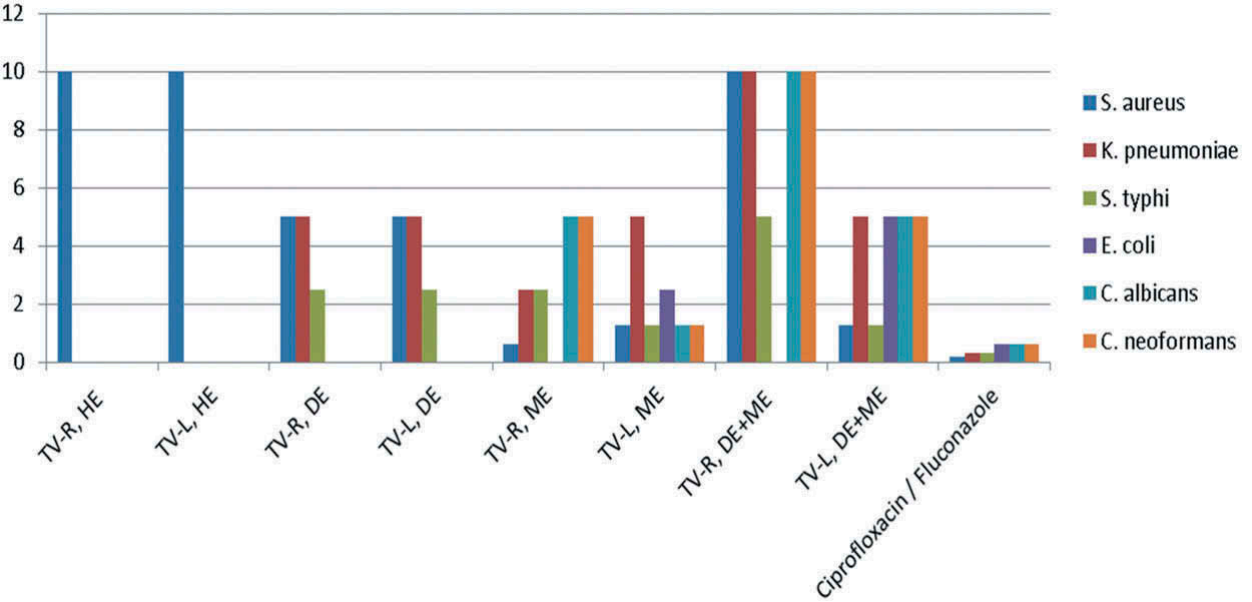
Graph showing MICs (Y-axis) against extracts and positive control (X-axis). In drawing, the MICs of extracts greater than 10 mg/mL were neglected by programme. **Key**: TV-R, HE = *Tephrosia vogelii*, root hexane extract; TV-L, HE = *Tephrosia vogelii*, leaf hexane extract; TV-R, DE = *Tephrosia vogelii*, root dichloromethane extract; TV-R, ME = *Tephrosia vogelii*, methanolic root extract; TV-R, DE+ME = *Tephrosia vogelii* roots, mixed dichloromethane and methanol extracts; TV-L, DE = *Tephrosia vogelii*, leaf dichloromethane extract; TV-L, ME = *Tephrosia vogelii*, leaf methanolic extract; TV-L, DE+ME = *Tephrosia vogelii* leaves, mixed dichloromethane and methanol extracts.

Minimum Inhibitory Concentrations (MICs) unveiled the extent of antimicrobial activities of the extracts against the tested microorganisms. The MICs varied from low to high against tested strains (Table 1 and Figure 2). The smaller MIC value indicates higher growth inhibitory activity of a given extract against the strains and vice versa (Eloff 1998). The *n*-hexane and dichloromethane extracts of *Tephrosia vogelii* leaves and roots exhibited low activities against fungal strains because their MICs were higher than 10 mg/mL. These low activities may be attributed to non-polar and less polar compounds present in the *n*-hexane and dichloromethane extracts. These activities signpost that the compounds contained in the latter extracts weakly inhibited growth of the fungi strains as compared to methanolic extracts.

**Table 1.** Antifungal and antibacterial activities of leaf and root extracts of *T. vogelii.*

Extracts and	Minimum Inhibitory Concentration (MIC) in mg/mL for bioassay tested species					
positive control	*S. aureus*	*K. pneumoniae*	*S. typhi*	*E. coli*	*C. albicans*	*C. neoformans*
TV-R, HE	10	˃10	˃10	˃10	˃10	˃10
TV-L, HE	10	˃10	˃10	˃10	˃10	˃10
TV-R, DE	5	5	2.5	˃10	˃10	˃10
TV-L, DE	5	5	2.5	˃10	˃10	˃10
TV-R, ME	1.25	2.5	2.5	˃10	5	5
TV-L, ME	1.25	5	1.25	2.5	1.25	1.25
TV-R, DE+ME	10	10	5	˃10	10	10
TV-L, DE+ME	1.25	5	1.25	5	5	5
Ciprofloxacin/Fluconazole	0.15625	0.3125	0.3125	0.625	0.625	0.625

Notably, root methanolic extracts of *T. vogelii* exhibited moderate activity at MIC of 5 mg/mL to both *C. albicans* and *C. neoformans*. Leaf methanolic extracts of *T. vogelii* exhibited a high potency due to its relatively low MIC of 1.25 mg/mL against both *C. albicans* and *C. neoformans*. Both fungal strains showed equally highly susceptibility to leaf methanolic extracts of *T. vogelii*. In principle, the methanolic extracts contain polar compounds such as phenolics, flavonoids, rotenoids and saponins which explain why the methanolic extracts demonstrated relatively higher bioactivities than other extracts (Mwaura et al. 2012; Stevenson et al. 2012; Swamy et. al. 2014; Stevenson and Belmain 2017). Moreover, it was interesting to note that the MIC of methanolic extracts was twice that of the positive control (fluconazole) suggesting superior inhibitory performance of the methanolic extracts on the fungi. The proximity of the MIC indicates that the compound(s) contained in the methanolic leaf extract have strong inhibitory effects on the fungi strains.

Moreover, we anticipated the blends; leaf dichloromethane with methanolic extracts (TV-L, DE+ME), and root dichloromethane with methanolic extracts (TV-L, DE+ME) in 1:1 ratio could exhibit synergistic effects against the tested fungi. Unfortunately, all extract blends showed antagonistic effects. For instance, antagonism was noted when MICs (Table 1) of sole methanolic extracts for both *T. vogelii* roots and leaves because the MIC values of the blends had considerably changed from small values (indicating high antifungal activity) to high values (indicating less antifungal activity). Although the blends showed antagonistic effects on both strains as compared to individual extracts, the potencies/inhibitory effects of the blend extracts from the leaves on these fungal strains were still superior to those from the roots. Apparently, the interactions of compounds in the mixed extracts reduce the inhibition power of the methanolic extracts for virtually more than half against the fungi strains.

Based on our findings, the antifungal activities of leaf and root methanolic extracts of *T. vogelii* against *C. albicans* and *C. neoformans* concur with other studies of the same plant that exhibited similar activities against bovine dermatophytosis (Makoshi and Arowolo 2011). Similarly, antifungal activities of methanolic extracts were in good alignment with other antifungal studies against pathogens causing human, animal and plant disease (Mahomoodally et al. 2008; Nneka and Jude 2012; Inalegwu and Sodipo 2015; Li et al. 2015). Thus, this study reports the leaf methanolic extracts and root methanolic extracts of *T. vogelii* that they are potential sources of antifungal agents against the opportunistic *Candida albicans* and *Cryptococcus neoformans* fungal strains. Nevertheless, these findings shed light to conduct toxicity, clinical tries, and structure characterisation of the potential antifungal compounds from the methanolic extracts of *T. vogelii.*

As for bioassays against the bacterial strains, the leaf and root hexane extracts of *T. vogelii* exhibited lowest activities on *Klebsiella pneumoniae, Salmonella typhi* and *Escherichia coli* because of high MICs (>10 mg/mL). Inhibitory effects of root hexane extracts on *Staphylococcus aureus* (Gram +ve bacteria) were evident at MIC of 10 mg/mL compared to Gram – ve bacteria. Presumably, unlike the Gram negative bacteria, lack of protective cell walls in *Staphylococcus aureus* might have allowed easy penetration of the bioactive agents of the extract into the bacterial cells, thereby increasing their susceptibility to root hexane extracts compared to their counterparts (Mahomoodally et al. 2008). The antibacterial activities of both root and leaf dichloromethane extracts of *T. vogelii* were moderate ranging from MIC 2.5 mg/mL against *S. typhi* to MIC 5 mg/mL against *S. aureus* and *K. pneumoniae*. However, the same extracts exhibited the lowest antibacterial activities (MIC > 10 mg/mL) against *E. coli* suggesting that the latter are more resistant strains to both dichloromethane root and leaf extracts. Perhaps, the compounds contained in the extracts are not chemically capable of disrupting the cell wall of *E. coli*. This might explain why the same compounds could express considerable activities against *K. pneumoniae* and *S. typhi*.

Of particular interest, to find out the synergistic effects of the blends against bacteria strains, the bioassays using mixtures of extracts (TV-L, DE+ME and TV-R, DE+ME) were performed. As a result, the mixture of root dichloromethane and methanolic extracts exhibited low antibacterial activities as compared to individual extracts. This could mean that the chemical interactions of compounds in the mixed extracts reduced efficacy against the bacterial strains. The activities of the mixed leaf dichloromethane and methanolic extracts demonstrated the performance in much the same way as individual methanolic extracts. Therefore, the blends of leaf extracts did not exhibit any positive synergistic effects on the bacterial growth instead antagonistic effects were more pronounced. Thus, it suggests that the interacting compounds in the mixed extracts could be suppressing antibacterial activities against the tested bacteria strains.

Besides, root methanolic extracts of *T. vogelii* exhibited highest activity with MIC 1.25 mg/mL against Gram-positive bacteria, *S. aureus* followed by *S. typhi* and *K. pneumoniae* at MIC 2.5 mg/mL. It exhibited low activity against *Escherichia coli* at MIC greater than 10 mg/mL. The leaf methanolic extracts of *T. vogelii* exhibited high antibacterial activities at MIC 1.25 mg/mL against *S. aureus* and *S. typhi*. It exhibited moderate at MIC of 2.5 mg/mL and 5 mg/mL against *E. coli* and *K. pneumoniae*, respectively. This observation is indicative of the presence of compound(s) in leaf extracts which inhibited *S. aureus, S. typhi* and *E. coli* but had less inhibitory effects on *K. pneumoniae*. Superbly, *S. aureus* and *S. typhi* were equally vulnerable to the methanolic leaf extracts of *T. vogelii*. The *E. coli* showed resistance to all extracts as they had high MIC greater than 10 mg/mL except leaf methanolic extracts to which it was susceptible at MIC of 2.5 mg/mL. Higher antibacterial activities of methanolic extracts of *T. vogelii* are attributed to polar compounds capable of suppressing the bacterial growth (Mahomoodally et al. 2008; Nneka and Jude 2012; Inalegwu and Sodipo 2015; Li et al. 2015). The antibacterial activities of ethanol-aqua extracts of *T. vogelii* barks reported by Swamy and co-workers are in good agreement with our findings that they are attributable to prospectively polar compounds as in leaf and root methanolic extracts (Swamy et al. 2014). Thus, the antibacterial activities obtained suggest that leaf and root methanolic extracts of *T. vogelii* are potential sources of antibacterial agents against the pathogens: *Staphylococcus aureus, Escherichia coli, Klebsiella pneumoniae* and *Salmonella typhi*.

## Conclusion

Our systematic bioassays of *Tephrosia vogelii* extracts proved that the plant extracts can potentially be used against fungal and bacterial diseases. The lower MIC values exerted by leaf extracts suggest that *Tephrosia vogelii* leaf plant material contains potent compounds against *Candida albicans* and *Cryptococcus neoformans*. Also, MIC suggest that the root and leaf methanolic extracts of *T. vogelii* contain potential and potent compounds which could be used to inhibit growth of *Staphylococcus aureus, Escherichia coli, Klebsiella pneumoniae* and *Salmonella typhi*.

Overall, our preliminary findings from antifungal and antibacterial activities of bioactives against *Candida albicans, Cryptococcus neoformans, Staphylococcus aureus, Escherichia coli, Klebsiella pneumoniae* and *Salmonella typhi* revealed that the leaf and root methanolic extracts of *T. vogelii* are potential source of antimicrobial agents for future development of pharmaceutical drugs. Moreover, these results shed light and should attract the attention to increase cultivation of *T. vogelii* for antifungal and antibacterial applications in addition to fishing applications. To ensure applicability of these findings, the future attention necessity focus on toxicity studies of the extracts/compounds, identification and characterisation of compounds from the bioactive extracts.
